# Exploring the Role of the Nucleus Accumbens in Adaptive Behavior Using Concurrent Intracranial and Extracranial Electrophysiological Recordings in Humans

**DOI:** 10.1523/ENEURO.0105-20.2020

**Published:** 2020-11-18

**Authors:** Nadine Eijsker, Guido van Wingen, Ruud Smolders, Dirk J. A. Smit, Damiaan Denys

**Affiliations:** 1 Department of Psychiatry, Amsterdam Neuroscience, Amsterdam UMC, University of Amsterdam, Amsterdam, 1105 AZ, The Netherlands; 2 Amsterdam Brain and Cognition, University of Amsterdam, Amsterdam, 1001 NK, The Netherlands

**Keywords:** cortico-striatal connectivity, electroencephalography, intracranial EEG, spectral power, stop signal task, θ oscillations

## Abstract

Recent human electrophysiological evidence implicated θ-band communication between the nucleus accumbens (NAc) and frontal and parietal cortex in cognitive flexibility. Since the NAc is connected with the motor system, we tested whether phase and amplitude-based NAc-cortical connectivity and power modulation likewise underlie flexibility in motor action control. We combined concurrently recorded intracranial and extracranial electroencephalograms from seven psychiatric patients implanted with deep brain stimulation (DBS) electrodes who performed a stop signal task (SST). Inhibition success, as opposed to failure, was associated with greater prestimulus information flow from right NAc to medial frontal cortex through phase coupling of θ oscillations. Inhibition failure evoked θ power increases in the left NAc and medial frontal cortex, whereas parieto-occipital cortex showed an α power decrease. We conclude that NAc-to-frontal θ connectivity, possibly facilitating processing of task-relevant information, and α and θ power modulations, possibly reflecting post-error engagement of cognitive control, contribute to adaptive behavior pertaining motor control.

## Significance Statement

Combining unique intracranial recordings from human nucleus accumbens (NAc) and concurrently recorded electroencephalographic (EEG) data, we complement previous research on the involvement of NAc-cortical θ-band communication in adaptive behavior by showing that prestimulus θ phase synchronization likely drives this process.

## Introduction

The nucleus accumbens (NAc) has a well-established role in reward processing and reinforcement learning (Cohen et al., 2009a, 2012; Lega et al., 2011; Patel et al., 2012). However, in recent years, it has additionally been implicated in cognitive flexibility (Floresco et al., 2006; van Schouwenburg et al., 2010; Yawata et al., 2012; Horschig et al., 2015). Specifically, interplay between the NAc and prefrontal cortex seems important for flexibility. The prefrontal cortex is thought to exert cognitive control by strategy development and active maintenance of goal-relevant representations (Miller and Cohen, 2001) and projects directly to the NAc, whereas the NAc seems to actively gate such task-relevant information (van Schouwenburg et al., 2010; Horschig et al., 2015) and indirectly projects back to frontal and parietal cortex via the globus pallidus, subthalamic nucleus and the medial dorsal nucleus of the thalamus (Alexander et al., 1986; Haber et al., 1995; Haber and Knutson, 2010). This idea was previously corroborated and extended using human intracranial electrophysiological data to show that the NAc increased θ-band communication to the neocortex, primarily frontal cortex and additionally parieto-occipital cortex, on processing of visual stimuli in a task requiring an attentional switch (Horschig et al., 2015). Moreover, these regions communicated with the NAc in the α-band during anticipation of visual processing.

Considering its connections, the NAc is seen as a functional link between the limbic and motor systems (Mogenson et al., 1980), yet the previously employed tasks only probed cognitive flexibility. Electrophysiological evidence for the involvement of the subthalamic nucleus, part of the motor system via which the NAc projects to the cortex, has already been found on a stop signal task (SST; Ray et al., 2012), a frequently adopted paradigm to study the ability to inhibit an ongoing motor response in the face of changing demands (response inhibition). Based on the NAc’s involvement in cognitive flexibility and its connections to the motor system, we tested whether the NAc is likewise involved in flexibility that pertains motor action directly. We likewise adopted the SST, which requires balancing speed (rapid response to a go-signal initiating action) and accuracy (successful inhibition of an ongoing response following a stop signal). The task is theoretically grounded in the horse-race model (Logan and Cowan, 1984), which posits that response inhibition depends on the relative finishing times of independent and competing go and stop processes. However, it may not be that simple; studies have found stimulus detection and action selection and execution to be influenced by both proactive and reactive control processes, with responses often being slowed to balance accuracy and speed (Bissett and Logan, 2011).

NAc-targeted deep brain stimulation (DBS) in compulsive and depressed patients offers the unique opportunity of recording intracranial electroencephalography (iEEG) from the human NAc and surrounding area. Since bidirectional cortico-striatal communication seems essential for optimization of flexible behavior, we combined intracranial and surface EEG recordings from psychiatric patients to investigate amplitude and phase-based cortico-striatal communication and power modulation during SST performance. If the role of the NAc in behavioral flexibility is similar to that in cognitive flexibility, we expect (1) α-band connectivity, specifically information flow from the cortex toward the NAc, during anticipation of stimulus presentation; followed by (2) θ-band connectivity, specifically information flow from the NAc toward the cortex, during stimulus processing.

## Materials and Methods

### Participants

Ten treatment-refractory psychiatric patients were recruited from the Academic Medical Center outpatient clinic. Two participants were excluded based on performance; one successfully inhibited on over 90% of stop trials, whereas the other completely lacked successful stop trials, resulting in too few trials in the remaining condition to analyze. Another participant displayed extreme amounts of β-band oscillations because of brain tumor removal. Of the remaining seven participants (aged 22–63 years; five females and two males), four were diagnosed with obsessive-compulsive disorder (OCD; one wth comorbid obsessive-compulsive personality disorder), two patients with major depressive disorder, and one patient with cocaine and opiate addiction (Table 1). All participants were right-handed and took their standard medication, with the exception of selective serotonin reuptake inhibitors. Medications included Euthyrox 50 mcg/d, Omeprazole 40 mg/d, Simvastatin 20 mg, Triazolam 100 mg/d, Suboxone 4 mg/d, Flucloxacilline 1000 mg/four daily, Nifedipine 40 mg/d, Selokeen 50 mg/d, Omeprazole 20 mg/d, Melatonin 5 mg/d, Promethazine 25 mg/d, Lorazepam 2,5 mg/d, Seroquel 300 mg/d, Parnate 30 mg/two daily, Domperidon 10 mg/d, and Movicolon and Paracetamol where necessary.

**Table 1:** Subject information and SST performance

ID	Sex	Age	Diagnosis	SSRT	Mean SSD	Mean RT correct go	Mean RT failed inhibition	% Successful inhibition	% Incorrect go
Patient 1	F	40	OCD	238	653	925	840	58	1
Patient 2	F	22	OCD	291	164	473	400	46	0
Patient 3	F	32	OCD	305	138	433	412	44	0
Patient 4	F	31	OCD	233	587	853	744	60	2
Patient 5	F	63	MDD	308	271	625	541	53	13
Patient 6	M	55	MDD	179	667	840	732	52	2
Patient 7	M	37	SUD	232	354	606	486	53	0
Summary mean (SD)	5 F/2 M	40 (14.3)		255 (47.9)	405 (228.4)	679 (194.9)	594 (176.7)	52% (5.6)	2% (4.6)

SSRT, stop signal reaction time; SSD, stop signal delay; RT, reaction time; F/M, female/male; OCD, obsessive-compulsive disorder; MDD, major depressive disorder; SUD, substance use disorder; SD, standard deviation.

The local Medical Ethical Committee of the Academic Medical Center approved the experiment and all participants provided written informed consent before the experiment.

### Stop signal task

#### Stimulus presentation

Stimuli were presented using Presentation (version 14.5; Neurobehavioral Systems) on a 15.4-inch laptop (HP 6730b) screen, placed ∼60 cm from the participants, at a resolution of 1024 by 768 pixels and with a refresh rate of 60 Hz.

#### Task properties

The SST consists of two types of trials. In go trials, a green arrow (go stimulus) pointing either to the left or right signals participants to press the corresponding, left or right, shift button on a keyboard as fast as possible using their left and right index fingers, respectively. In stop trials, the arrow changes color from green to red (stop stimulus) after a variable delay; the stop signal delay (SSD). This signals participants to cancel their motor response to the go stimulus. Participants were instructed to respond to go signals as fast as possible, while simultaneously minimizing inhibition failures, and that these two criteria were equally important.

Our task consisted of three blocks of 100 trials each, of which 70% go trials and 30% stop trials. The intertrial interval, during which a fixation cross was presented, varied randomly between 1750, 1875, 2000, 2125, and 2250 ms, with each interval presented equally often. Go stimuli were presented until response or a stop stimulus appeared, with a maximum of 1200 ms. The SSD started at 300 ms and was increased and decreased with 50 ms after every successful and failed inhibition trial, respectively, for the left and right-hand side independently. This double staircase procedure thus increased inhibition difficulty, by increasing the amount of time between go and stop stimuli, after successful inhibition and vice versa after failed inhibition, which steers toward generating approximately equal numbers of successful and failed inhibition trials. The SSD was not reset between blocks.

### Data acquisition

#### iEEG recordings

Patients were bilaterally implanted with deep brain electrodes (Medtronic model 3387) in the NAc between 2010 and 2012. Stereotactical placement of the electrodes was performed as previously described by van den Munckhof et al. (2013), which included planning based on T1-weighted magnetic resonance images, online measurement over the electrodes to inform when the gray matter target was reached, and subsequent confirmation with a postoperative CT scan. Each electrode contained four contact points, each being 1.5 mm in length and separated by 0.5 mm. The most ventral contact point was positioned in the NAc core, with the other contact points extending into the ventral part of the anterior limb of the internal capsule. Our sample performed the SST on day 4 after surgery for implantation of the deep brain electrodes, except for patient 7, who was tested on the fifth day after surgery. Patients would later undergo surgery for implantation of the stimulator.

#### EEG recordings

EEG was recorded at a sampling rate of 512 Hz using a 64-channel recording system with shielded Ag/AgCl electrodes (Advanced Neuro Technology B.V.) following the international 10–10 system. Of the 64 channels, eight (four per electrode) were used for the iEEG and four for collecting horizontal and vertical eye-movement. No signals were recorded from the areas covered by postsurgery bandages. Data were online common average referenced.

### Data analysis

#### Behavioral performance

Performance measures were calculated over all trials available before artifact rejection. Stop signal reaction time (RT) was calculated using the quantile method (Verbruggen and Logan, 2009), which is less susceptible to violations of assumptions underlying the horse-race model than other methods (Band et al., 2003; Verbruggen and Logan, 2009). Per individual, this included calculating the quantile RT (QRT), which is the correct go trial RT (sorted ascendingly) corresponding to the quantile of the proportion of failed stop trials, and subsequently subtracting the mean SSD.

#### Preprocessing

**Figure 1.**
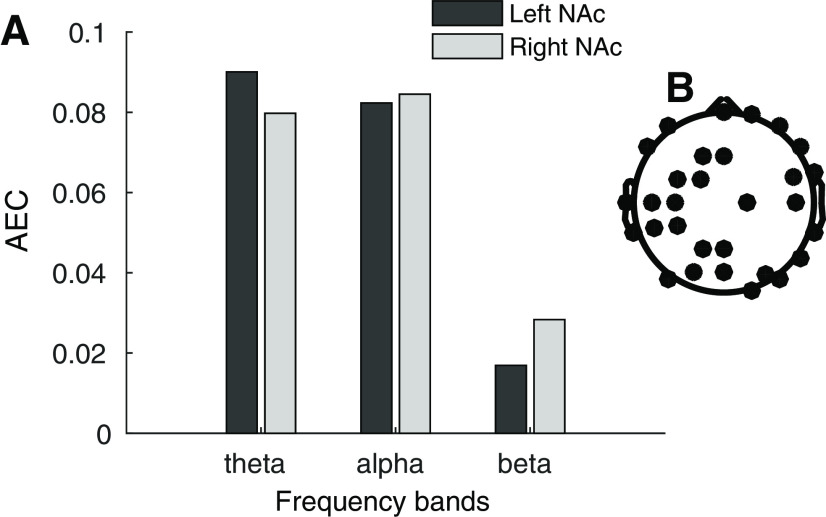
AEC in the θ (4–8 Hz), α (8–13 Hz), and β (13–30 Hz) frequency bands on the overall task. ***A***, The bars depict average AEC between the NAc (most ventral contact point of the DBS electrode) and 30 randomly selected surface EEG channels, for the left and right NAc separately. ***B***, Topology of the 30 randomly selected surface electrodes.

Data were preprocessed using the EEGLAB-toolbox (version 14.1.1; Delorme and Makeig, 2004) in MATLAB (version R2014b; The MathWorks). Signals with a SD below 10% of the median or >10 times the median, for EEG and iEEG signals separately, were considered flatlines and too noisy, respectively, and rejected. Then, signals were re-referenced to the average of the respective signal type, i.e., EEG and iEEG. For iEEG, this was done separately for the left and right hemispheres. Signals were bandpass filtered (FIR filter with Hamming window) between 1 and 47 Hz and down-sampled to 256 Hz. The latter was done to improve performance of the EEGLAB-plugin Automatic Artefact Rejection (Gomez-Herrero et al., 2006), using canonical correlation analysis algorithms for blind source separation, which was adopted for automatic removal of muscular artefacts in the EEG signals only. Subsequently, EEG signals were visually inspected and channels considered too noisy were rejected, after which they again were re-referenced to the mean of remaining channels. Then, we extracted epochs from −1000 to 1200 ms relative to go stimulus onset, which we corrected for baseline activity and visually inspected to reject epochs containing artifacts (all except eye blinks). We used principal component analysis to reduce data dimensionality to 45 components (with the exception of 42 components for one patient with <45 EEG channels at this point) and exclude minor components. We then ran an independent component analysis on the remaining signals and rejected components containing eye blinks and other noise. We then extracted two subsets of data; from −550 to 550 ms relative to stop signal onset (stop trials only) and to motor response (correct go and failed inhibition trials). For the latter, we excluded trials that contained multiple motor responses and randomly selected an equal number of go trials to keep the number of trials equal between conditions, as there was less failed inhibition than go trials available per participant. We did not do this for the stop trials, because of their limited numbers. Based on previous literature (Cohen et al., 2009b; Horschig et al., 2015), we filtered all signals (using a FIR filter with Hamming window) in the θ (4–8 Hz) and α (8–13 Hz) frequency ranges. To check for potential relevance of β oscillations, we looked at overall task β connectivity. For this, we calculated amplitude envelope correlation (AEC), an amplitude-based connectivity measure that allows for some variability in frequency between signals (further description below), for the θ, α, and β (13–30 Hz) frequency bands between bilateral NAc and 30 randomly selected surface EEG channels on overall task data (per subject an average of 256 trials of all conditions combined, lasting from −500 to 2200 ms relative to the go signal). We then compared these frequency bands using a one-way ANOVA (*F*_(2,18)_ = 4.12, *p* = 0.034) and subsequent two-sample *t* tests, which indicated significantly less connectivity in the β-band, compared with the θ-band (*t*_(12)_ = 2.7, *p* = 0.019) and α-band (*t*_(12)_ = 2.97, *p* = 0.012). Figure 1 depicts AEC on the overall task per frequency band, averaged over the NAc, surface EEG channels pairs, for the left and right NAc separately, as well as the topology of the selected surface electrodes. Based hereon, β-band oscillations were excluded from further analyses.

#### Connectivity measures

We have adopted two complementary connectivity measures that rely on different oscillatory characteristics to detect coupling between anatomically distributed sources: amplitude and phase. First, AEC detects coupling based on correlated amplitude modulations, thought to reflect the extent of synchrony of neural assemblies (Varela et al., 2001), thus independently of presence of phase coherence and differences in frequency. This method was found a suitable complementary measure to coherence for detecting longer-range, polysynaptic, cortical γ interaction in humans (Bruns et al., 2000) and subcortical-cortical β-γ coupling in LFPs recorded in cats (Bekisz and Wróbel, 1999). We calculated AEC by correlating the Hilbert envelopes of the signals. Second, directed phase transfer entropy (dPTE) estimates the direction of information flow using transfer entropy between instantaneous phase time-series. It was implemented as described in detail by Lobier et al. (2014), who showed that it quantifies directed connectivity in a model-free manner that is robust to realistic amounts of noise and linear mixing. First, timeseries were complex filtered using the Hilbert transform, then the phase angle was extracted from the complex signals using angle, which were put in a range of 0–2*π. The phases were binned using the number of bins defined in Scott (1992). These binned phases were compared with phases of the second signal after a predefined lag of ∼10 ms. Since lag precision was restricted by sampling rate, ultimately, approximation of the 10-ms interaction lag was 11.7 ms. Transfer entropy, which is based on the principle that, if signal X influences signal Y, the probability density of signal Y’s future conditioned on its past should differ from that conditioned on the pasts both signals X and Y (Schreiber, 2000), with the probability density quantified by Shannon Entropy (Shannon, 1948). Lastly, dPTE was normalized using the marginal probability densities (i.e., within signal transfer entrophies), resulting in values ranging between 0 and 1, with 0.5–1 indicating influence of X over Y, 0–0.5 indicating influence of Y over X, and 0.5 indicating absence of preferential direction of information flow. We used MATLAB to implement dPTE. We calculated both connectivity measures over the entire trial lengths to optimize the accuracy of the low frequency phase estimates, considering their strong dependence hereon.

#### Power spectral density (PSD)

We calculated PSD separately for the 550 ms before and after the event (stop signal onset or motor response). To this end, we first applied a fast Fourier transform. Then, to calculate PSD in decibel (dB), we used this Fourier transformed data as input for the following formula: 10*log10(((1/(srate*sum(slength))) * *abs(F)*.^2)*2), where F is the Fourier transformed data, srate is the sampling rate, and slength is the number of samples in the signal. With a frequency resolution of 1.8 Hz, we averaged the PSD at ∼3.6, 5.4, and 7.2 Hz for the θ frequency PSD and 9, 10.8, and 12.6 Hz for the α frequency PSD. From here on out, we will refer to PSD as power.

#### Statistical analyses

We employed three levels of correction in this descriptive study. First, to account for the dependency across trials within subject, we applied linear mixed-effects modeling (LMM; MATLAB’s *fitlme*) with random effects for subject. Second, to correct for the total number of channels tested and account for non-normality, we employed 10,000-iteration permutation tests with maximum and minimum *t* distributions. This is a method generally used to control the family wise error rate in neuroimaging research, yet it is also suitable for electrophysiological data (Kilner et al., 2005). Third, we Bonferroni-corrected for the number of frequency bands, connectivity measures, and hemispheres tested. We tested the most ventral iEEG channel per hemisphere, located in the NAc, and EEG channels that previously showed connectivity with the NAc during cognitive flexibility (Horschig et al., 2015). These were channels Fp1, Fpz, Fp2, AF7, F7, F1, Fz, FCz, P1, Pz, P2, POz, PO4, O1, and O2. Since subject 6 lacked usable signal from the right NAc, analyses on this channel are based on six instead of seven participants.

For the connectivity analyses, the LMM included random effects for subject and a fixed effect for condition. We tested the connectivity measures separately and over the entire epoch, as opposed to pre. Per iteration of the permutation test, we first randomly shuffled the condition labels within subject before fitting the LMM. The labels were identically shuffled for θ and α-filtered signals. Then, we took the maximum and minimum *t* values across all channels to form the null distributions. For dPTE, the 2.5th percentile of the minimum *t* distribution and the 97.5th percentile of the maximum *t* distribution constituted the critical values for the lower and upper tails, respectively, consistent with two-tailed testing. Because dPTE is a directed measure, testing both tails reflects testing for both cortex-to-NAc and NAc-to-cortex communication. For AEC, the 95th percentile of the maximum *t* distribution constituted the critical value for the upper tail, consistent with one-tailed testing. This reflect testing for coupling, but not decoupling, of signals. Ultimately, we Bonferroni-corrected for four comparisons (two frequency bands × two connectivity measures), resulting in a critical *p* value of 0.0125.

For the power analyses, the LMM included random effects for subject and fixed effects for condition, time, and the interaction between condition and time (pre vs post event). We shuffled the condition labels within subject and time-period (pre or post event), formed null distributions of the maximum and minimum *t* values per iteration and performed two-tailed tests as described above. However, here, we tested the iEEG channels against their individual null distributions instead of being collapsed with the EEG channels (and corrected for this via Bonferroni correction), whereas the null distributions for the EEG channels were based on all EEG channels (identical to the connectivity analyses), thereby correcting for the number of channels tested. Ultimately, we Bonferroni-corrected the iEEG channels for four comparisons (two frequency bands × two hemispheres) and the EEG channels for two comparisons (two frequency bands), resulting in critical *p* values of 0.0125 and 0.025, respectively.

#### *Post hoc* testing

Significant condition effects in connectivity were subjected to *post hoc* testing to inform about the timing of the found effect. This included calculating the relevant connectivity measure for a 500-ms sliding window with a stepsize of 23.4 ms, resulting in 26 time windows. For stepsize, we approximated 25 ms, yet precision was restricted by sampling rate. Solely for visualization purposes, we interpolated missing electrodes using spherical spline interpolation (EEGLAB toolbox). For visualization of the sliding window analysis, we oversampled (factor 5) and smoothed (two-point moving average, i.e., 10-point for the oversampled data) the data.

To see whether significant condition effects in connectivity were specific to the most ventrally located contact point of the DBS electrode (L/R0), targeted at the NAc, we tested whether the effect(s) could also be found on the most dorsally located DBS contact point (L/R3). As expected, when average referencing included R0, power spectra for R3 consistently showed lower power than when average referencing excluded R0. This suggests that the signal measured at L/R0 contains considerably higher spectral power. Therefore, we referenced L/R3 against all available other contact points of the DBS electrode (L/R1/L/R2) except for L/R0. For just comparison, we repeated the 10,000-iteration permutation test, using the signals from L/R3 instead of L/R0 to calculate connectivity with the surface electrodes. Lastly, considering hemispheric lateralization of motor planning and execution (Sabate et al., 2004), we checked for lateralization of significant connectivity results by adding a main effect of side (left/right trial) and its interaction with condition to the LMM and applying this to the relevant channel pair(s).

For significant power results, we calculated the percentage of change in power over time using the following formula: (10̂(diff/10) −1)*100, where diff is the difference in grand average from pre to post event. Additionally, we tested whether α and θ power changes were related on a trial-by-trial basis by applying LMMs on the pre-to-post power changes with random effects for subject. We also tested whether significant power modulations were specific to the most ventrally located contact point of the DBS electrode (L/R0) or could also be found on the most dorsally located contact point (L/R3). L/R3 was tested against its own maximum *t* distribution, resulting from a 10,000-iteration permutation. Lastly, we checked for lateralization of power modulation by adding a main effect of side (left/right trial) and its interactions with condition and time (pre or post event) to the LMM and applying this to the relevant channel pair(s).

## Results

### Task performance

Table 1 shows sample characteristics and behavioral performance. On average, participants showed an SSRT of 255 ms, indicative of the time required to inhibit an already initiated motor response, they successfully inhibited their response in 52% of stop trials, and either failed to respond or responded incorrectly in 2% of go trials. Mean RTs on correct go and failed inhibition trials were 679 and 594 ms, respectively. The former seems considerably longer and somewhat more variable than generally reported for both healthy participants and OCD patients (Penadés et al., 2007; Boisseau et al., 2012). However, whereas OCD patients usually show longer SSRTs than controls (Lipszyc and Schachar, 2010), current SSRT lies within the ranges reported for both controls and patients; seemingly somewhere in between their means, yet the literature shows considerable variability. Notably, the depressed participants showed the most omissions (13% and 2%) on go trials.

### Connectivity between NAc and the cortex

**Figure 2.**
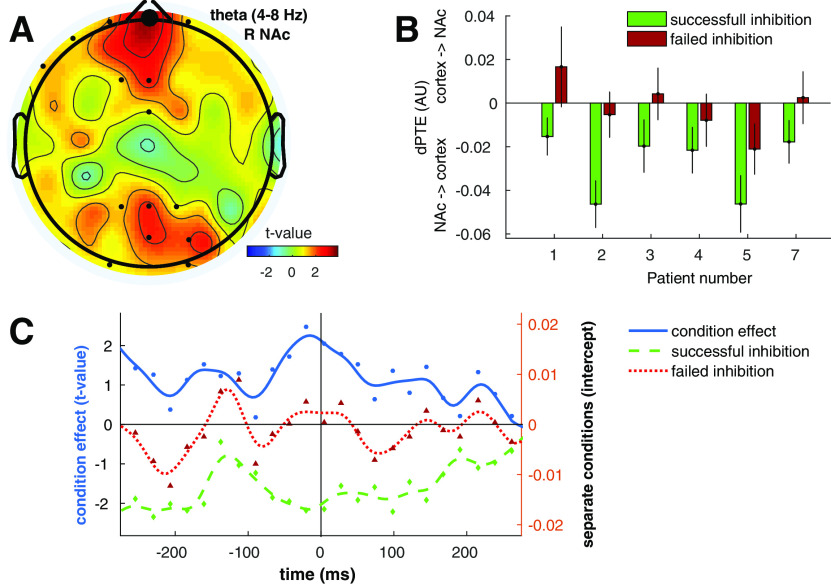
Effect of inhibition success on dPTE between right NAc and scalp electrodes. ***A***, dPTE between right NAc and EEG electrode Fpz (large dot) showed a condition effect in the θ-band on successful versus failed inhibition trials (−550−550 ms relative to stop stimulus onset). LMM *t* values are plotted with small dots indicating tested channels. ***B***, Mean dPTE (arbitrary units, centered) for conditions and patients separately. Positive and negative values indicate cortex→NAc and NAc→cortex information flow, respectively. Error bars indicate SEM. ***C***, *Post hoc* sliding window analysis showed the effect was highest just before stop stimulus onset (time = 0). Condition effect *t* values (solid line) were smoothed and plotted on the left *y*-axis, whereas the right *y*-axis reflects centered smoothed dPTE intercepts (dashed lines) for the separate conditions, with negative values again indicating effective connectivity from the NAc toward the cortex and vice versa. Since dPTE was calculated for a sliding window, with each dot representing 500 ms, the approximately −290−290 ms shown on the *x*-axis represents the entire −550- to 550-ms trial length.

When comparing connectivity during the −550–550 ms relative to stop signal onset between successful and failed inhibition trials, we found that inhibition success was associated with more negative dPTE between the right NAc and Fpz in the θ-band (Fig. 2*A*; Table 2). This effect was stable across subjects (Fig. 2*B*). *Post hoc* sliding window analysis revealed that this effect, which reflects information flow from the NAc to Fpz, was already present before stop stimulus onset (Fig. 2*C*). This effect was not different for left versus right trials (*t*_(462)_ = 0.73, *p* = 0.466), nor did it show an interaction effect between condition and side (*t*_(462)_ = 0.28, *p* = 0.779). Furthermore, θ-band dPTE between R3, the most dorsally located DBS contact point, and Fpz did not show a significant condition effect (*t*_(464)_ = 2.52, *p* = 0.073, *p*-Bonferroni-corrected = 0.291), suggesting that the effect is local to R0, the most ventrally located DBS contact points, targeted at the NAc. No effects were found for AEC or connectivity in the α-band.

**Table 2:** Channel availability

ID	Intracranial contact points rejected^a^	EEG channels missing from selection	Number of EEG channels rejected	Number of EEG channels not recorded
Patient 1	R1	AF7	3, including AF7	9
Patient 2	R2	AF7	1, including AF7	9
Patient 3	R1	O1, Oz, O2	6, including O1, Oz, O2	8
Patient 4	R2	Fp2	6	8
Patient 5	R1		0	6
Patient 6	R0, R1	AF7	10, including AF7	10
Patient 7			4	8

a R = right hemisphere, 0 = most ventral contact point, located in the NAc, 1–2 = contact points one and two places, respectively, more dorsal from the most ventral contact point/NAc.

When comparing connectivity during the −550–550 ms relative to motor response between failed inhibition and correct go trials, we did not find any effects after Bonferroni correction. Before correction for four comparisons, we saw more positive AEC between the right NAc and O1 in the θ-band (*t*_(464)_ = 2.76, *p* = 0.0442) and more positive dPTE between the left NAc and P1 in the α-band (*t*_(519)_ = 3.21, *p* = 0.022; Table 3) on failed inhibition compared with correct go trials.

**Table 3:** Condition effects in connectivity between NAc and the cortex

Time-locking: conditions	Connectivity measure	Frequency band	NAc hemisphere	EEG	*t* value	*p* value before Bonferroni correction	*p* value after Bonferroni correction
Stop: successful vs failed inhibition	dPTE	θ	R	Fpz	−3.70	0.0030	0.0120
Response: failed inhibition vs correct go^*^	AEC	θ	R	O1	2.76	0.0442	0.1768
	dPTE	α	L	P1	3.21	0.0220	0.0880

* Solely significant before Bonferroni correction.

**Table 4:** PSD modulation following motor response on failed inhibition versus correct go trials

Frequency band	Effect	Channel	*t* value	*p* value before Bonferroni correction	*p* value after Bonferroni correction
θ	Condition × time	NAc L	3.29	0.0004	0.0016
	Condition × time	FCz	3.94	0.0006	0.0012
α	Condition	NAc L	1.87	0.0284	0.1136^*^
	Condition × time	PO4	−3.11	0.0110	0.0220

* Solely significant before Bonferroni correction.

### Power modulation in the NAc and the cortex

When comparing power between successful and failed inhibition trials and changes between the 550-ms pre versus post stop signal onset, we did not find significant effects for condition or the interaction between time and condition after Bonferroni correction. Neither θ nor α power modulation around stop signal onset seems to underlie inhibition success.

**Figure 3.**
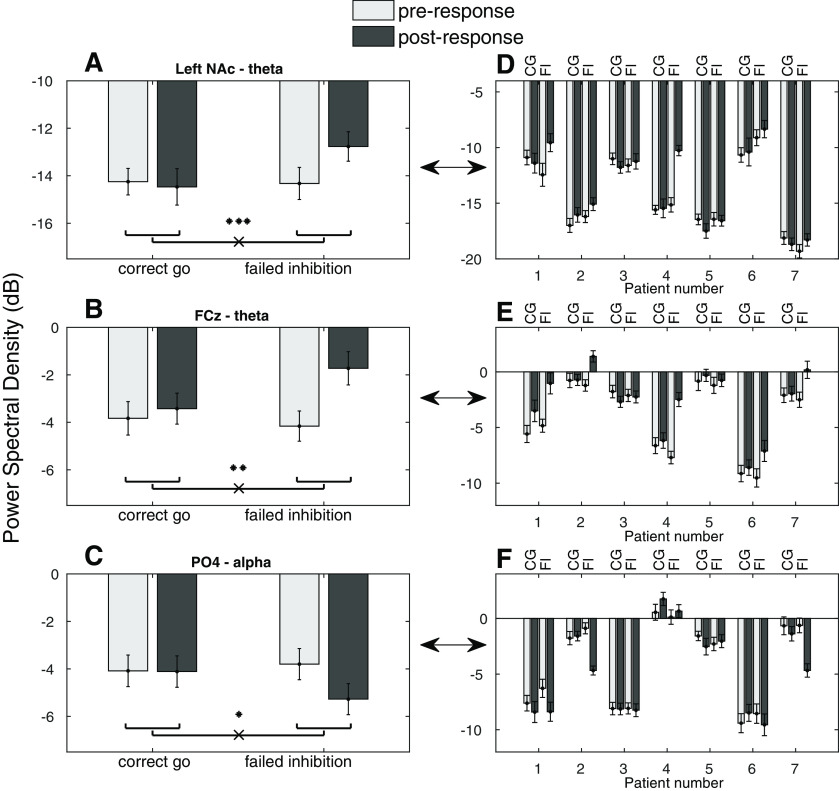
PSD modulation following response on failed inhibition versus correct go trials. Power is expressed in decibel (dB). Error bars indicate SEM. Greater θ power increase following motor response on failed inhibition compared with correct go trials (***A***) in the left NAc and (***B***) on electrode FCz. ***C***, Greater α power decrease following motor response on failed inhibition compared with correct go trials on electrode PO4. ***D–F***, Power modulation from plots ***A–C***, respectively, visualized for patients separately. CG and FI refer to correct go and failed inhibition conditions, respectively. *** *p* < 0.0005 before and 0.002 after Bonferroni correction, ** *p* < 0.001 before and 0.005 after Bonferroni correction, * *p* < 0.05.

When comparing power between failed inhibition and correct go trials and changes between the 550-ms pre versus post motor response, we found a greater θ power increase after response on failed inhibition trials compared with correct go trials in the left NAc and at electrode FCz (Fig. 3*A*,*B*; Table 4). This was accompanied by a greater decrease in α power at electrode PO4 (Fig. 3*C*). These changes in power from pre-to-post response on correct go and failed inhibition trials, respectively, were 5% and 43% for the left NAc, 10% and 75% for FCz, and 1% and 29% for PO4. Figure 3*D–F* shows the variability of these effects over subjects. We found no lateralization of θ power modulation in the left NAc (side: *t*_(872)_ = 0.44, *p* = 0.657; side × condition × time: *t* = 1.73, *p* = 0.083) or FCz (side: *t* = 0.11, *p* = 0.911; side × condition × time: *t* = 0.13, *p* = 0.894), nor of α power modulation on PO4 (side: *t* = 0.43, *p* = 0.669; side × condition × time: *t* = 0.40, *p* = 0.689). Before Bonferroni correction for four comparisons, we additionally found an effect of condition on α power in the left NAc, showing 13% more α power on failed inhibition compared with correct go trials (*t*_(876)_ = 1.87, *p* = 0.0284). *Post hoc* LMMs indicated no significant linear relationship between the changes in α power at PO4 and θ power in the left NAc (*t*_(218)_ = −1.6, *p* = 0.1138) or at FCz (*t*_(218)_ = −1.47, *p* = 0.1430). Specificity analysis showed a significant, yet somewhat smaller, interaction effect on θ power at the most dorsally located contact point of the left DBS electrode (*t* = 3.26, *p* = 0.0007, *p*-Bonferroni-corrected = 0.0028). The effect showed a similar pattern to that found on the most ventrally located contact point, with pre-to-post response power changes of −1% and 41% on correct go and failed inhibition trials, respectively.

## Discussion

We found that inhibition success, as opposed to failure, was associated with increased information flow from right NAc to medial frontal cortex through phase coupling of θ oscillations, present already before stop signal onset. We additionally found that θ power increased following motor response on failed inhibition compared with correct go trials in both the left NAc and medial frontal cortex, whereas parieto-occipital cortex showed an α power decrease.

To our knowledge, this is the first report to show involvement of the NAc and its communication with frontal cortex in adaptive behavior pertaining motor control. Lack of significant findings for AEC or in the α-band suggests θ phase specificity of NAc-frontal cortex communication underlying inhibition success. However, some trends suggested the possibility of θ amplitude coupling between right NAc and (left) occipital cortex and/or α phase coupling between the left NAc and (left) parietal cortex to distinguish between correct going and failed inhibition.

The observed connectivity is consistent with the finding of NAc-to-frontal cortex θ-band granger causality during anticipation of a visual stimulus during a task of cognitive flexibility (Horschig et al., 2015). We likewise found that communication was already present before, and sustained around, stimulus presentation. Computational models suggest that the ventral striatum might actively gate sensory information based on task demands maintained in frontal regions of cognitive control (Frank et al., 2001). This idea was previously substantiated by showing that the NAc modulated fronto-parietal coherence in the α-band, which is in line with a nonlinear dynamic causal modeling fMRI study showing that shifts in attention relied on the ventral striatopallidum to modulate connectivity between stimulus-specific visual association areas and the prefrontal cortex (van Schouwenburg et al., 2010). Therefore, the currently found prestimulus phase coupling between NAc and frontal cortex might likewise reflect facilitation of task-relevant information. Considering the visual SST, this information likely originates from visual cortex and flows via the globus pallidus, subthalamic nucleus, and thalamus to frontal cortex (Haber et al., 1995; Haber and Knutson, 2010). In line with this view, we found a trend toward θ amplitude coupling between right NAc and electrode O1. These findings extend the well-established role of the NAc in reward processing and reinforcement learning (Cohen et al., 2009a, 2012; Lega et al., 2011; Patel et al., 2012) to the context of adaptive behavior in tasks of both cognitive and behavioral flexibility.

However, we did not observe the poststimulus increase in information flow from the NAc to the cortex that was previously found (Horschig et al., 2015). This might be explained by task differences, since stimulus presentation in the former task required redirection of attention and a subsequent button press, whereas it required inhibition of a button press in the present task. In addition, it previously was unclear whether the effects relied on phase or amplitude locking, because granger causality depends on both such signal components (Lobier et al., 2014). We now extend those findings by showing that the information flow from the NAc to the cortex depends on θ phase synchronization, rather than amplitude coupling. In contrast to other intracranial studies that found connectivity from the cortex to the NAc during attentional switching and reward anticipation, we did not find information flow from the cortex to the NAc, being indicative of cognitive control, to underlie inhibition success (Cohen et al., 2012; Horschig et al., 2015).

We additionally found an increase in midfrontal and left NAc θ power as well as a decrease in parieto-occipital α power after response on failed inhibition compared with correct go trials. θ Power increases in both NAc and medial frontal cortex have previously been found following, especially negative, feedback on a variety of tasks (Cohen et al., 2007, 2009b; Münte et al., 2008; Nurislamova et al., 2019). The well-established feedback or error-related negativity, evoked by erroneous (motor) responses, has been found to arise from a combination of a power increase and partial phase synchronization of θ oscillations (Luu et al., 2004; Trujillo and Allen, 2007). These signals have been traced back to the anterior cingulate cortex and/or pre-SMA, which are involved in error and conflict processing and subsequent behavioral adjustment (Garavan et al., 2002; Luu et al., 2004; Iannaccone et al., 2015). Interestingly, it has been postulated that, whereas high θ might reflect the conflict monitoring process itself, low θ underlies the more general process of interregional communication and thus relays the error to other areas of cognitive control (Huster et al., 2013). Current power increases were more pronounced for lower θ frequencies, especially so for electrode FCz, pointing to engagement of cognitive control after failed inhibition. Performance monitoring-related θ power increases often co-occur with increased θ phase synchronization between medial frontal and parietal cortex (Nurislamova et al., 2019), which was previously found to be modulated by the NAc during attentional switching (Horschig et al., 2015). Although we found a concurrent decrease in posterior α power, something previously found to accompany midfrontal θ increases following failed inhibition on a Go/NoGo task (Mazaheri et al., 2009), these θ and α power modulations were not significantly related on a trial-by-trial basis. However, with *p* = 0.11 and *p* = 0.14 for those relationships, this might have resulted from our limited sample size. α Power increases are thought to decrease local neural processing capacity, thereby inhibiting a region’s activity (Jensen and Mazaheri, 2010). Therefore, our α power decrease might reflect release of inhibition on the posterior parietal regions involved in action planning and decision-making, likely resulting from top–down influence of the frontal control system (Andersen and Cui, 2009).

In conclusion, our results supplement current knowledge about cortical involvement in performance monitoring by implicating NAc θ power modulation in the engagement of cognitive control after inhibition failure, possibly for subsequent adjustment of decision-making parameters to prevent additional errors. This extends the previous finding that subthalamic nucleus θ power and coherence with frontal cortex are likewise modulated during the SST (Alegre et al., 2013). Similar to the θ power increases we found in the NAc and frontal cortex and relate to the well-established error-related negativity, the subthalamic nucleus showed inhibition failure-related increases in θ power and coherence with frontal cortex. If and how communications between these subcortical structures underlies feedback processing remains unanswered, yet these findings point to a role of θ oscillations herein. Additionally, we found inhibition success-related θ connectivity between the NAc and frontal cortex that was absent in the subthalamic nucleus. Although the inhibition success-related connectivity was specific to the most ventrally located contact point of the right DBS electrode, targeted at the NAc, we found the postresponse θ power increase also on the most dorsally located contact point of the left DBS electrode. Although all targeted at the NAc, slight differences in DBS electrode location between patients could explain this non-specificity, considering that the medially located contact points were used as reference. Alternatively, it could be that the power change is not restricted to the gray matter of the NAc. Lack of significant lateralization of our main effects substantiates their interpretation as higher-order regulatory rather than primary motor processes (Sabate et al., 2004).

Notwithstanding the unique dataset, it comes with some limitations. First of all, although we allowed for random effects for subject in our models, the sample size of seven subjects limits statistical power and generalizability of the results. To account for this and limit (unnecessary) multiple comparison correction, we tested a selection of EEG channels based on previous research (Horschig et al., 2015), yet thereby limiting exploration of potentially unexpected findings. Also, we were unable to measure the impedances of the DBS electrode contact points, informative of signal quality, since doing so could potentially induce non-therapeutic stimulation. Furthermore, we must keep in mind that our results might represent pathologic brain functioning, since we used a severely affected psychiatric sample and lacked a control group. Especially so since NAc-DBS is thought to exert its therapeutic effects through targeting NA-cortical connectivity (Figee et al., 2013; Smolders et al., 2013). Also, MDD patients show aberrant error-related negativity (Tucker et al., 2003; Holmes and Pizzagalli, 2008), which has been linked to midline frontal θ oscillations during action regulation (Luu and Tucker, 2001). However, cortical feedback-related negativity has been found not to differ between controls and DBS-implanted OCD and Tourette’s syndrome patients (Schüller et al., 2015) and we found stable and significant results in a sample that included various disorders, albeit mainly disorders of compulsivity. Moreover, participants were not taking SSRIs at the time of data collection, yet postsurgical analgesics could have affected brain functioning.

We found condition-specific phase-synchronization and power modulation for separate time periods of task performance, yet associations between oscillatory phase and power, a phenomenon called cross-frequency coupling, have additionally been reported. Coupling of γ power to α phase in the NAc was found during reward processing (Cohen et al., 2009a), decreased before strategic switching (Cohen et al., 2009b), and differentiated between positive and negative feedback (Lega et al., 2011). Moreover, NAc γ-θ coupling varied with cognitive control during a motor learning task (Dürschmid, 2013). To gain more insight into the interplay between subcortical and cortical local cross-frequency coupling and phase synchronization between distant regions, such associations should be tested directly using datasets such as ours. Additionally, since increased θ phase synchronization between bilateral NAc has been linked to behavioral adjustment following losses (Cohen et al., 2009b), inter-NAc connectivity might also be relevant for SST performance. Lastly, the possibility of functional hemispheric differentiation of the NAc warrant further investigation, considering we found right lateralized inhibition success-related connectivity changes and left lateralized performance monitoring-related power changes.

In sum, because of our unique dataset of concurrent striatal and EEG recordings, we were able to show involvement of prestimulus NAc-to-medial frontal cortex θ phase synchronization in successful response inhibition and both cortical and NAc power modulation in the θ-band and α-band in performance monitoring on the SST. These results corroborate earlier findings that θ oscillations are crucial for cortical-subcortical communication during cognitive processing and involvement of the NAc in adaptive behavior. However, still plenty remains to be learned about both the specificity and the extent of interplay of different features of oscillatory activity, including cross-frequency coupling and the relationship between NAc-cortical communication and cortical interactions.

## Synthesis

Reviewing Editor: Daniela Schiller, Mount Sinai School of Medicine Decisions are customarily a result of the Reviewing Editor and the peer reviewers coming together and discussing their recommendations until a consensus is reached. When revisions are invited, a fact-based synthesis statement explaining their decision and outlining what is needed to prepare a revision will be listed below. The following reviewer(s) agreed to reveal their identity: Allison C Waters, Florian Mormann.

Advance to the field:

This manuscript provides an important cross-domain generalization (from cognitive to motor) of theta band communication between nucleus accumbens and frontal cortex in behavioral control processes.

This study extends previous research on the involvement of nucleus accumbens-cortical theta-band communication in adaptive behavior by showing that prestimulus theta phase synchronization likely drives this process.

Software Comments:

EEGLAB-toolbox and Matlab are software packages in common usage. The functions identified in the manuscript are standard in the field. Not enough information was provided to comment on software used to calculate connectivity measures

Manuscript comments:

Review #1

The central finding (NAc to cortex dPTE with successful motor inhibition) is novel and important. Although the sample size was small, this effect was observed on the level of individuals. The combination of scalp and intracranial recording provides a rare and valuable insight into functions that involve cortical-subcortical connectivity via phase synchronization. The secondary finding (midline theta increase following errors) may be less novel when interpreted in the context of theta contributions to the error-related negativity. Statistical analyses were appropriate for the research question and controlled for multiple comparisons. The manuscript was a pleasure to read and adequately referenced.

A few ideas for revision:

1. A strength of the manuscript is generalization from cognitive to motor control. With this in mind, I would have expected analysis of beta band activity, which is key to understanding movement disorders and STN DBS treatment mechanisms, in addition to / instead of alpha.

2. Sliding window analysis shows largest effect before the stimulus, but present throughout the epoch. It's not clear that this is a motor function, per se, or attention in the context of a motor task. These ideas are explored in the discussion but could also be addressed with some minor changes in emphasis elsewhere in the manuscript (e.g., abstract).

3. STN vs NAc. A key piece of rationale for the analysis of NAc to frontal cortex was the work of Alegre et al., 2013, who showed STN power/coherence with PFC on same SST task. A more systematic discussion of divergence/convergence with those findings could strengthen the interpretation. Another idea would be to run a post-hoc control analysis on the dorsal-most contact to test for specificity of the effect to the presumed NAc target. The dorsal-most contact would vary in location (probably in the anterior internal capsule somewhere) depending on the lead trajectory. Would a null result lend credit to the idea that this is really a NAc-specific effect?

Minor comments:

-Clarify procedures when and where testing occurred relative to implantation; implications for confounding effects of anesthesia.

-As interpretation hinges on NAc target, describe method for surgical targeting and confirmation of target engagement in the NAc (e.g. CT warped to atlas?). With such a small sample, it would also be feasible to provide a figure showing the location of the ventral contacts in MNI space (left and right).

-Reported hemispheric specificity is interesting. Where there also effects of hemisphere in the impedances of the ventral-most contact?

-It may be useful for future elaborations on this work in more homogenous samples to list the medications.

-Clarify how LFP was referenced. To the IPG? To the average of the EEG?

-Though a reference is provided, the paper would be strengthened by an explanation of directed phase transfer entropy as a connectivity measure, as well as information about software, functions or code used in the analysis.

-EEG channel selection is a little strange. Though drawn from a previous study, the channels selected for analysis are asymmetrical over frontal cortex. What channels were interpolated because of bandages?

-Although result were not clearly divergent in the MDD patients, an effect of depression status has been shown in midline negativities and midline theta. This could be noted in addition to the null effects in Tourette and OCD.

Review #2

The paper “Exploring the role of the nucleus accumbens in adaptive behavior using concurrent intracranial and extracranial electrophysiological recordings in humans” reports on extra- and intracranial electrophysiological recordings in seven psychiatric patients implanted with depth electrodes in the nucleus accumbens (NAc). The authors investigated adaptive behavior pertaining motor control by having subjects perform a stop signal task. Using amplitude envelope correlation and directed phase transfer entropy as connectivity measures, they report right lateralized inhibition success-related connectivity changes and left lateralized performance monitoring-related power changes. They conclude that NAc-to-frontal theta connectivity, possibly facilitating processing of task-relevant information, and alpha and theta power modulations, possibly reflecting post-error engagement of cognitive control, contribute to adaptive behavior pertaining motor control.

The data analyzed in this study are rare and difficult to obtain. The methods are sophisticated and sound. The paper is very well written and addresses a topic potentially helpful to understand the neurophysiological mechanisms of motor control. Nonetheless, I have a few concerns that should be addressed before publication.

Major points:

(1) Laterality analysis.

In the behavioral task, subjects were instructed to press a left or right button. How was the motor response executed? Were the two buttons pressed using the same finger? Two different fingers of one hand? Two different hands? Since the present study is specifically designed to investigate motor control and flexibility (as opposed to cognitive control and flexibility), the contralateral topology of motor function and planning should be taken into account. If button presses were performed using different hands, it would be interesting to see a separate analysis of left vs. right trials. If it was just one hand, a separate left vs. right analysis would still be informative, as would be a direct comparison of activity in the contralateral brain hemisphere in comparison to the ipsilateral one.

(2) Cross-frequency coupling analysis.

Towards the end of the Discussion, the authors write “To gain more insight into the interplay between subcortical and cortical local cross-frequency coupling and phase synchronization between distant regions, such associations should be tested directly using datasets such as ours.” I completely agree, but I don’t understand what’s keeping the authors from actually doing this? They have the data and obviously also the expertise to perform such an analysis. I realize this will require a bit of additional work, but it would render the current study far more complete. I wonder if the authors could be persuaded to include this analysis.

Minor points:

(1) Nomenclature.

The term local field potential (LFP) should be reserved for micro-electrode signals only. The cylindrical contacts used on clinical depth electrodes capture the activity of hundreds of thousands of neurons. Compared to micro-electrode signals, depth electrode signals are simply not local and should therefore be referred to as field potentials or intracranial EEG (iEEG). The authors should check this throughout the manuscript.

(2) Preprocessing.

The authors state that signals that were considered flatlines or too noisy we excluded from the analysis. How many channels were these? This number, or better the number of electrodes actually analyzed per patient, should be listed in Table 1.

(3) Referencing.

Authors state that “signals were re-referenced to the mean of the remaining channels for EEG, left-hemisphere LFP, and right-hemisphere LFP signals separately”. Does this mean there were three different references, one for left NAc, one for right NAc, and one for all the scalp EEG channels? Why did the scalp EEG channels not receive separate references per hemisphere when the NAc channels did?

(4) Preprocessing.

Authors state “We ran an independent component analysis [...] using principal component analysis”. Since ICA and PCA are two different approaches, the authors should be clear about which of the two they used.

(5) Statistical Analyses.

The authors calculated the percentage of change in power as 10^(diff/10)-1)*100. I’m not sure I understand this formula. Was this applied to the power values in decibles?

Typos.

-p.17, top: “to underlying inhibition success”: Did you mean “to be underlying inhibition success” or “to underlie inhibition success”?
